# Estrus Detection Using Background Image Subtraction Technique in Tie-Stalled Cows

**DOI:** 10.3390/ani11061795

**Published:** 2021-06-16

**Authors:** Shogo Higaki, Kei Horihata, Chie Suzuki, Reina Sakurai, Tomoko Suda, Koji Yoshioka

**Affiliations:** 1 National Institute of Animal Health, National Agriculture and Food Research Organization, Tsukuba, Ibaraki 305-0856, Japan; higakis668@affrc.go.jp (S.H.); schie@affrc.go.jp (C.S.); sakurair972@affrc.go.jp (R.S.); sudatomoko@affrc.go.jp (T.S.); 2 Kyushu Okinawa Agricultural Research Center, National Agriculture and Food Research Organization, Kōshi, Kumamoto 861-1192, Japan; horihatak850@affrc.go.jp; 3 Laboratory of Theriogenology, School of Veterinary Medicine, Azabu University, Sagamihara, Kanagawa 252-5201, Japan

**Keywords:** background subtraction, cattle, estrus detection, precision agriculture, tie-stall housing

## Abstract

**Simple Summary:**

With increasing herd sizes and labor costs in recent decades, visual estrus detection by farmers has become more difficult because of the reduced manpower input per cow. To address this problem, various wearable devices have been developed for automatic estrus detection in cows, such as neck- or leg-mounted activity meters for monitoring estrus-associated increments in the amount of activity. However, these animal-contact devices have several limitations; namely, it can be dangerous to attach or remove the device and it can cause discomfort. Recently, a background image subtraction technique has been proposed as a non-contact method for monitoring activity changes in livestock animals. In this study, a new method was developed by combining the background subtraction technique and the thresholding method to detect estrus-associated activity increases in tie-stalled cows. Using this method, a substantial increase in activity in estrus was detectable, and the estrus detection sensitivity reached as high as 90% with a precision of 50%, where the sensitivity and precision were calculated as: (true-positive/[true-positive + false-negative]) × 100% and (true-positive/[true-positive + false-positive]) × 100%, respectively. This result may indicate that activity monitoring using the background subtraction technique has the potential to be a non-contact estrus detection method in tie-stalled cows.

**Abstract:**

In this study, we determined the applicability of the background image subtraction technique to detect estrus in tie-stalled cows. To investigate the impact of the camera shooting direction, webcams were set up to capture the front, top, and rear views of a cow simultaneously. Video recording was performed for a total of ten estrous cycles in six cows. Standing estrus was confirmed by testing at 6 h intervals. From the end of estrus, transrectal ultrasonography was performed every 2 h to confirm ovulation time. Foreground objects (moving objects) were extracted in the videos using the background subtraction technique, and the pixels were counted at each frame of five frames-per-second sequences. After calculating the hourly averaged pixel counts, the change in values was expressed as the pixel ratio (total value during the last 24 h/total value during the last 24 to 48 h). The mean pixel ratio gradually increased at approximately 48 h before ovulation, and the highest value was observed at estrus, regardless of the camera shooting direction. When using front-view videos with an appropriate threshold, estrus was detected with 90% sensitivity and 50% precision. The present method in particular has the potential to be a non-contact estrus detection method for tie-stalled cows.

## 1. Introduction

Visual estrus detection by cattle farmers has become more difficult because of the reduced manpower input per cow due to increasing herd size and labor costs in recent decades [1]. To address this problem, various wearable devices have been developed for automatic estrus detection in cattle [2]. The most common and successful devices are neck- or leg-mounted activity meters (accelerometer and pedometer) for detecting estrus-associated increments in the amount of activity. However, most of the available devices are focused on untethered cows, such as cows under free-stall housing and at pasture. Furthermore, these animal-contact devices have several limitations; for example, they can be dangerous to attach or remove and can cause discomfort.

Computer vision has been proposed as a non-contact information acquisition method for livestock behavior analysis [3]. Background image subtraction is a widely used method for detecting foreground objects (moving objects) in videos by subtracting the current video frame from the background model (static objects). Hence, the activity level of animals can be monitored from the frame-to-frame changes in the sum of the foreground pixels. This approach has been applied to detect activity changes in sows during final gestation [4]. However, it has never been used for estrus detection in cows. Therefore, in this study, we determined the applicability of the background subtraction technique to detect estrus-associated increments in activity in cows, particularly under tie-stall housing conditions.

## 2. Materials and Methods

### 2.1. Animals

Six cows (four nulliparous Holstein and two parous Japanese Black cows; 40–115 months old, 550–650 kg) that were non-pregnant, non-lactating, and previously had normal estrous cycles, were used. All cows were housed in tie-stalls (approximately 1 m wide by 1.8 m long) under natural temperature conditions, and they were fed twice daily with hay and concentrate to meet the Japanese Feeding Standard recommendations. The experiment was conducted at the National Institute of Animal Health, National Agriculture and Food Research Organization, Japan, from September 2019 to June 2020.

### 2.2. Data Collection

**Figure 1:**
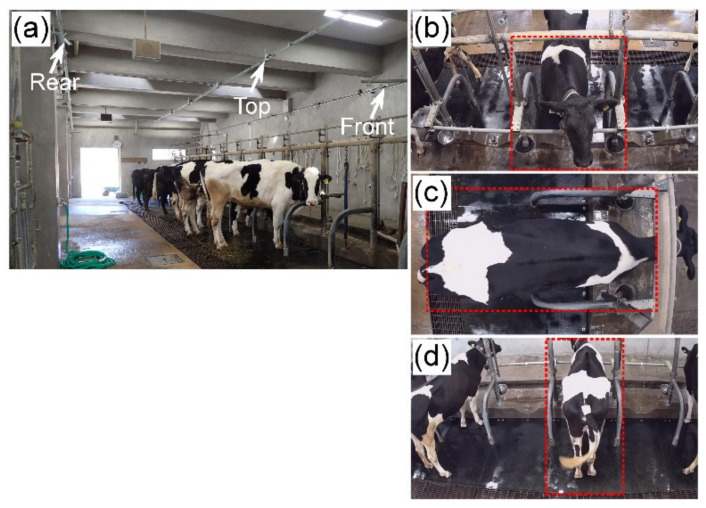
Position of webcams and example of captured frames. (**a**) Position of webcam. Three webcams were set up to capture the front, top, and rear views of the cow. The front-view webcam was placed 2.2 m ahead of the animal at a height of 3.3 m. The top-view webcam was placed 3.5 m above a tie-stall. The rear-view webcam was placed 2.7 m behind the animal at a height of 3.3 m. (**b**–**d**) Examples of frames captured by the front, top, and rear webcams, respectively. Red-dotted rectangles indicate regions of interest (510 × 640, 1000 × 540, and 350 × 720 pixels for the videos from the front, top, and rear webcams, respectively).

Cows during a total of ten estrous cycles, (each of the six animals was used one to three times) from day 10 of the cycle (day 0 = ovulation day) to day 11 of the subsequent cycle, were recorded by video. To investigate the impact of the camera shooting direction, internet protocol webcams (VStarcam C7816WIP, Shenzhen VStarcam Technology, Shenzhen, China) were set up to capture the front (at 2.2 m from the front and at 3.3 m above), top (at 3.5 m centrally from the top), and rear (at 2.7 m from the rear and at 3.3 m above) views of a cow simultaneously (Figure 1a). The camera was equipped with a 720P (1280 × 720 resolution pixels) complementary metal oxide semiconductor sensor and infrared illuminator, which provided infrared illumination to 15 m at low illumination such as experienced at night-time. Software (Eye4, Shenzhen VStarcam Technology) bundled with the camera was installed on a laptop computer to record videos. This system produced color sequences at 25 frames per second (fps) under optimal illumination and automatically switched to grayscale sequences (in the infrared spectrum) at approximately 10 fps under poor lighting conditions (<0.8 lx). Examples of the front-, top-, and rear-view images are shown in Figure 1b,c,d, respectively.

During the experimental period, the follicular and luteal structures in the ovaries were examined daily using transrectal ultrasonography. After the commencement of luteolysis, the tested cows were led out of the cowshed to a paddock and attempted to be mounted by herd mates at 6 h intervals to detect standing-to-be-mounted behavior (it took approximately 10 min). The onset and end of estrus were defined as 3 h before the time when a standing response was first observed and ceased, respectively. From the end of estrus, ultrasonography was performed every 2 h to confirm the ovulation time. Ovulation was defined as the time when the largest follicle in the ovary had disappeared, and it was assumed to occur 1 h before the disappearance of the follicle.

### 2.3. Video Data Analysis

**Figure 2:**
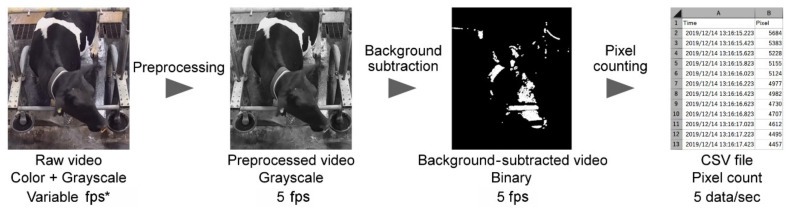
Video data analysis workflow. In the preprocessing step, all videos were converted to grayscale at a constant frame rate (5 fps). In the background subtraction step, the foreground (white pixels: moving objects) was extracted from each frame using the k-nearest neighbor-based background/foreground segmentation algorithm with the morphological opening operator. In the pixel-counting step, foreground pixels in a region of interest were counted at every frame. * Frames per second.

As shown in Figure 2, the recorded videos were analyzed in the following three steps: preprocessing, background subtraction, and pixel counting. In the preprocessing step, all videos were converted to grayscale at a constant frame rate (5 fps) to minimize the effects of color information and variable frame intervals using an open source video transcoder (HandBrake, Ver. 1.2.2) [5]. The background subtraction step was performed using a computer vision library (OpenCV, Ver. 3.4.9) [6] with the k-nearest neighbor-based background/foreground segmentation algorithm [7]. A morphological opening function of the library, which includes erosion followed by dilation, was applied to reduce the scattered noise pixels. The results from the background subtraction step were treated as binary images, where 0 represents the background (black pixels: static objects), whereas 1 represents the foreground (white pixels: moving objects). In the pixel-counting step, foreground pixels (white pixels in the background-subtracted video) in a region of interest, which were clipped out of the sequences (510 × 640, 1000 × 540, and 350 × 720 pixels for the images from the front, top, and rear webcams, respectively), were counted at every frame, at a rate of 5 data per second.

### 2.4. Background-Subtracted Pixel Data Analysis

To reduce the influence of biasing factors in the videos (such as artificial light changes, presence of farm staff, and absence of animals for estrus confirmation), the hourly averaged pixel counts were calculated and used for subsequent analysis. To exclude the influence of the circadian rhythm, changes in hourly averaged pixel counts were expressed as the pixel ratio (total pixel counts during the last 24 h/total pixel counts during the last 24 to 48 h). Theoretically, the pixel ratio becomes constant at 1 (baseline) if there is no daily fluctuation in the pixel counts. However, there were several short-term (within a few hours) inconsequential ups and downs in the ratios; hence, the exponentially weighted moving average (EWMA) was applied to smooth the data. EWMA was calculated using the following formula:

EWMA(0) = X(0)

EWMA(t) = αX(t) + (1 − α) EWMA(t − 1), and t > 0.

where EWMAt denotes the EWMA at time “t,” X_t_ the measured value at time “t,” and EWMA_t-1_ the EWMA at time “t − 1.” Parameter α was set to 0.1.

### 2.5. Estrus Detection based on Background-Subtracted Pixel Data

To determine the estrus-associated increase in pixel ratio, five thresholds were set based on the standard deviation (SD) from within the cow mean of the pixel ratios during the preceding period, namely, 1.6 SD, 1.7 SD, 1.8 SD, 1.9 SD, and 2.0 SD above the baseline. When the pixel ratios exceeded the threshold for more than three consecutive hours, we regarded the increments as estrus alerts. Estrus alerts observed during the estrus period were regarded as true positive (TP) predictors. Non-alerted estrus events and alerted non-estrus events were regarded as false negatives (FN) and false positives (FP), respectively. Sensitivity and precision were calculated as: (true positive/[true positive + false negative]) × 100 (%) and (true-positive/[true-positive + false-positive]) × 100 (%), respectively. The F_1_ score, the harmonic mean of sensitivity and precision, was calculated as 2 × sensitivity × precision/(sensitivity + precision). Optimal detection of estrus was considered to occur when the F_1_ score was the highest.

### 2.6. Statistical Analysis

Hourly values of pixel ratio from 168 h prior to 240 h after ovulation were compared with the values during the reference period (−240 to −169 h from ovulation: mid-luteal phase) using the Steel test. Statistical analyses were performed using the KyPlot software (Ver. 5.0; KyensLab, Tokyo, Japan), and differences were considered significant when the *p*-value was < 0.05. Values are presented as the mean ± SD unless otherwise specified.

## 3. Results

The mean duration of video recording was 26.2 ± 5.6 days. Standing-to-be-mounted behavior was observed in all ten estrous cycles with the starting and ending times at −29.8 ± 5.1 h and −12.5 ± 4.0 h from ovulation, respectively.

### 3.1. Changes in Pixel Ratio during Estrous Cycle

**Figure 3:**
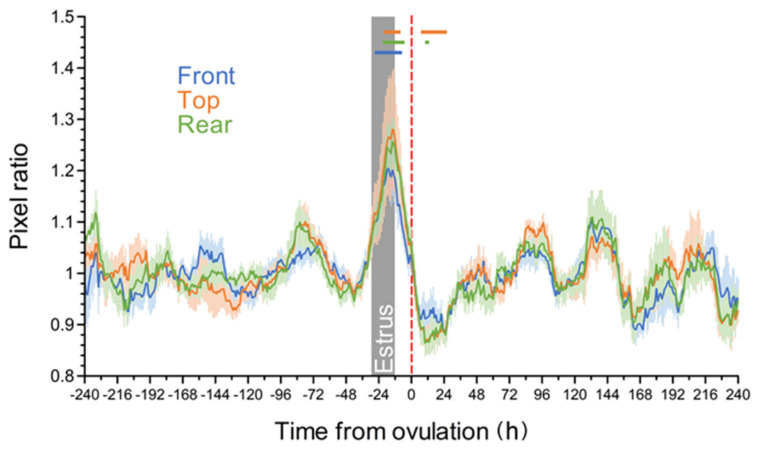
Changes in pixel ratio during a natural estrous cycle in tie-stalled cows. The pixel ratio was calculated as the total value during the last 24 h/total value during the last 24 to 48 h. Blue, orange, and green horizontal bars indicate the time points with significant differences between values at the indicated time point and the corresponding mean values during the reference period (−240 h to −169 h from ovulation) (*p* < 0.05, Steel test). The gray vertical bar indicates the mean period of standing estrus (from 29.8 to 12.5 h before ovulation). The data were standardized for ovulation (0 h: red dotted vertical line). Data are expressed as mean ± standard error. Each line includes the data of ten estrous cycles.

As shown in Figure 3, the mean pixel ratio gradually increased at approximately −48 h from ovulation. The values near estrus were higher than those at the reference period (−240 h and −169 h from ovulation) (*p* < 0.05), with a peak at approximately −20 h from ovulation, regardless of the camera shooting direction.

### 3.2. Estrus Detection based on Background-Subtracted Pixel Data

Estrus detection sensitivities and precisions both varied with camera shooting directions and thresholds for the pixel ratios (Table 1). Under the tested conditions, the highest F_1_ score (0.64) was achieved by setting the threshold of 1.8 SD for the videos captured by the front webcam. Using these detection criteria, the mean intervals from the start and end of estrus alert to ovulation were 19.3 ± 5.2 h and 4.6 ± 5.8 h, respectively. TP, FP, and FN events, shown in Table 1, were distributed almost equally across all tested animals (data not shown).

## 4. Discussion

In this study, we determined the applicability of the background subtraction technique to detect estrus in tie-stalled cows. Using this technique, a substantial increase in the pixel ratio during estrus could be detected, regardless of the camera shooting direction. Based on the pixel ratio, the F_1_ score of estrus detection reached as high as 0.64, with a sensitivity of 90.0% and precision of 50.0%. To the best of our knowledge, this is the first report on the use of the background subtraction technique for estrous detection in cows under tie-stall housing.

In tie-stalled cows, estrus detection mainly relies on visual observation by the farmer, and the detection sensitivity and precision were approximately 50% [8] and 80% [9], respectively. Although the precision of the present method was lower than that of visual observation, the higher sensitivity of the present method might be advantageous. In estrus detection in cattle farming, false negatives (non-alerted estrus events), which resulted in the extension of the calving interval by approximately 21 days (one estrous cycle) by mistakenly eliminating estrous cows, should be more problematic than false positives (alerted non-estrus events), whereas false positives may lead to a relatively limited amount of extra labor needed for confirming estrus under tie-stall conditions.

The estrus detection performance of the present method was comparable to that of previous studies using neck- or leg-mounted activity meters in tie-stalled cows; sensitivity and precision were 67–92% and 34–83%, respectively [10,11,12]. These studies focused on identifying the day of estrus or the 48 h time window before ovulation rather than a more specific period. To improve fertility, an automatic estrus detection system must be highly correlated with the optimal insemination time, 6–30 h before ovulation [13,14]. The present estrus detection alerts might allow cows to be artificially inseminated within a suitable time window, owing to timely estrus detection at approximately 19 h before ovulation (ranging from 13 to 28 h). As a further advantage, the present method is completely free from the risks of attaching/detaching the device and discomfort.

The superiority of using the front-view video could be consistent with the findings of a previous study that determined the impact of pedometer locations (attached to the neck, front leg, and hind leg) on estrus detection performance [12]. Estrus-associated increments in activity were more pronounced in the front leg, hind leg, and neck, resulting in the highest estrus detection potency of the front leg pedometers in tie-stalled cows [12]. The background subtraction technique in particular, preferably with a front-view video, could be used to detect estrus in tie-stalled cows.

## 5. Conclusions

Videos were recorded for a total of ten estrous cycles in six cows in tie-stall housing. Foreground objects (moving objects) were extracted in the videos using the background subtraction technique, and the foreground pixels were counted at every frame of the video sequence and converted to grayscale at a constant frame rate (5 fps). Based on the background-subtracted pixel data, a substantial increase in activity during estrus was detected, and the F_1_ score of estrus detection reached as high as 0.64, with a sensitivity of 90% and precision of 50%. However, it should be noted that the present method was validated on ten estrous cycles of six cows. Various factors are known to affect the intensity and duration of estrus, such as parity, milk production, and body condition score [15]. Hence, a follow-up study with a larger sample size is required.

## 6. Patents

Drs. Higaki and Yoshioka have a patent pending for the use of the background subtraction technique for estrus detection in tie-stalled cows (Japanese patent pending, No. 2020-126539).

## Figures and Tables

**Table 1:** Estrus detection performance based on background-subtracted pixel data.

Camera Position	Threshold	True Positive	False Positive	False Negative	Sensitivity (%)	Precision (%)	F_1_ Score
Front	1.6 SD	9	14	1	90.0	39.1	0.545
	1.7 SD	9	11	1	90.0	45.0	0.600
	1.8 SD	9	9	1	90.0	50.0	0.643
	1.9 SD	7	8	3	70.0	46.7	0.560
	2.0 SD	7	6	3	70.0	53.8	0.609
Top	1.6 SD	8	15	2	80.0	34.8	0.471
	1.7 SD	7	14	3	70.0	33.3	0.452
	1.8 SD	7	10	3	70.0	41.2	0.519
	1.9 SD	7	8	3	70.0	46.7	0.560
	2.0 SD	7	6	3	70.0	53.8	0.609
Rear	1.6 SD	8	12	2	80.0	40.0	0.533
	1.7 SD	8	11	2	80.0	42.1	0.552
	1.8 SD	7	11	3	70.0	38.9	0.500
	1.9 SD	7	10	3	70.0	41.2	0.519
	2.0 SD	6	10	4	60.0	37.5	0.462

The sensitivity, precision, and F1 score were calculated as TP/(TP + FN) × 100, TP/(TP + FP) × 100, and 2 × sensitivity × precision/(sensitivity + precision), respectively, where TP denotes true positive, FN false negative, and FP false positive.

## Data Availability

The datasets generated during and/or analyzed during the current study are available from the corresponding author on reasonable request.
